# Atomized droplet size prediction for supersonic atomized water drainage and natural gas extraction

**DOI:** 10.1038/s41598-022-26597-x

**Published:** 2022-12-23

**Authors:** Chengting Liu, Liang He

**Affiliations:** 1 School of Petroleum Engineering, Northeast Petroleum University, Daqing, 163318 China; 2 Key Laboratory of Enhanced Oil Recovery (Northeast Petroleum University), Ministry of Education, Da qing, China

**Keywords:** Computational science, Mechanical engineering, Natural gas, Software

## Abstract

In the later stage of natural gas reservoir exploration, the wellbore pressure is reduced and the liquid accumulation is serious, in order to solve the problem of liquid accumulation and low production in low-pressure and low-yield gas wells, the supersonic atomization drainage gas recovery technology is used to improve the recovery rate. By studying the influence of working condition parameters of downhole nozzle atomization drainage gas recovery on atomization effect and liquid carrying rate, a new physical model of atomization nozzle is established, the back propagation (BP) neural network atomization model and BP neural network atomization model optimized by genetic algorithm (GA) is established, and the Matlab is used to train the 45 groups of data sets before the experiment. After the model training, the normalized atomization parameters are trained for sensitivity analysis. The relationship between the strength and weakness of the factors affecting Sotel's average droplet particle size (SMD) is as follows: gas flow (*Q*_*g*_) > liquid inlet diameter (*d*) > liquid phase flow (*Q*_*l*_). The last 15 sets of data sets outside the training samples were tested by BP model and BP neural model optimized by genetic algorithm (GA-BP), and the size of SMD was predicted. The experimental results show that the determination coefficient R^2^ of the established GA-BP network model to the experimental parameters is 0.979 and the goodness of fit is high; the mean square error (MSE), mean absolute error (MAE) and mean absolute percentage error (MAPE) of the predicted value of GA-BP atomization model and the experimental value are 4.471, 1.811 and 0.031 respectively, the error is small, the prediction accuracy is high, and the establishment of the model is accurate. The GA-BP model can efficiently predict SMD under different operating conditions, at present, the new supersonic atomizing nozzle has been successfully applied to the Xushen gas field block of Daqing Oilfield, which can improve the recovery rate of natural gas by 4.5–8.6%, alleviate the problem of effusion near the end of oil exploration, and has certain guiding significance for solving the problem of wellbore effusion and improving production efficiency.

## Introduction

In the later stage of natural gas well production, the bottom hole pressure decreases, the gas flow velocity decreases, the liquid carrying rate decreases, the wellbore liquid accumulation increases, the liquid accumulation hinders the natural gas migration, and the production decreases. The liquid discharge efficiency of traditional technologies such as gas lift, bubble discharge, natural gas circulation and high pressure pump is poor^1–4^. In order to solve the problem of serious fluid accumulation in wellbore, a new technology of nozzle atomization for drainage and gas recovery is adopted^5–7^. The technology uses the energy of natural gas to atomize the liquid accumulated in the bottom hole through the nozzle, and the droplets are carried out of the wellbore along with the natural gas. This method reduces energy consumption, improves energy utilization, reduces the operation and maintenance cost of gas wells, and can effectively solve the problem of wellbore fluid accumulation.

Many scholars have carried out related research on nozzle atomization technology, carried out corresponding atomization experiments^8,9^, and achieved ideal results. Han et al.^10^ designed a kind of internal mixing atomization and dedusting nozzle. The flow field in the nozzle was simulated by FLUENT. The results show that with the increase of water supply pressure, the flow velocity in the nozzle increases, the air velocity decreases, and the gas–liquid relative velocity decreases. They carried out spray dust reduction experiments through atomizing nozzles. The experimental results show that when the water supply pressure increases, the range, droplet volume fraction and droplet size all increase, and the dust reduction efficiency of total dust and respirable dust increases at first and then decreases. KOMAG Mining Technology Research Institute^11^ has developed a kind of water spray nozzle, which can be effectively used to spray dust reduction at the transfer point of shearer, roadheader and conveyor. Feng^12^ carried out atomization drainage tests on low-pressure and low-production gas wells using nozzles at the production site of West Sichuan gas field. The atomization effect of nozzles is good, the liquid carrying rate of wellbore is increased by 23.4%, and the liquid production is increased by 200 m^3^/d. Ni et al.^13^ put the supersonic nozzle atomization device at the depth of 1000–2000 m to carry out field experiments, which shows that the outlet velocity is greatly higher than that of ordinary nozzles, and the liquid-carrying efficiency is increased by 41.3%.

In recent years, the experimental means have also been innovated due to the progress of science and technology, through the in-depth study of spray experiments, in order to obtain better atomization effect, so as to achieve better economic value. Benjanin et al.^14^ studied the internal and external flow field of swirl nozzle. According to the experimental results, the model formulas of mass flow coefficient and droplet velocity were obtained. Seoksu et al.^15^ studied the internal structure and static pressure of swirl nozzle, and the study showed that there was backflow vortex in the swirl atomization process, and the pressure drop inside the nozzle was larger under high injection pressure. Lee et al.^16^ carried out experiments on pass nozzles, and optimized the pass nozzles by CFD simulation, optimizing four design variables: nozzle inlet length, outlet length, inlet diameter and radial position. The results show that the optimized nozzle reduces the total pressure loss and increases the mass flow, and the optimized pre-swirl system reduces the aerodynamic loss, increases the mass flow rate under a certain pressure, and satisfies the pressure margin of blade cooling. The supersonic atomization nozzle model was established by Los Alamos^17^ laboratory in the United States, and the supersonic atomization combustion process of internal combustion engine was studied. The analysis of the complex motion of evaporation, fragmentation and turbulent diffusion of two-phase chemical fluid can effectively improve the efficiency of atomization combustion.

The atomization effect of the nozzle can be evaluated by studying the droplet size SMD and its distribution, which are the key parameters of the atomization performance. Lilan et al.^18^ studied the droplet size distribution in the flow field of external air atomizer through experiments. They divide the atomization field into several observation areas, and through the measurement of several local observation areas, the relationship of droplet size distribution in the whole atomization field is obtained, which provides a certain reference for the study of nozzle atomization field. it provides a basis for intuitively understanding the droplet size distribution of the nozzle atomization field. Yu et al.^19^ developed a new type of gasified coal water slurry nozzle and studied its atomization performance. They discussed in detail the effects of nozzle working load and gas flow rate on atomization particle distribution, Sauter mean diameter SMD and nozzle atomization angle. Hyun Suh et al.^20^ studied the effect of cavitation flow on the atomization characteristics of diesel fuel in different size nozzles through the flow visualization system, and used the particle measurement system to determine the atomization characteristics such as SMD and droplet average velocity. The results show that the cavitation in the nozzle enhances the fuel atomization performance, and the longer the nozzle orifice length is, the more fuel atomization is. Xia et al.^21^ used laser particle size system to measure the droplet diameter of different spray fields. It was found that the droplet size in the spray center was the smallest and the SMD at the spray edge increased.

The influence of working condition parameters on atomization effect was studied, and the experimental studies on the influence factors of different operating parameters on droplet diameter were carried out, some scholars have established corresponding mathematical models to predict SMD to improve atomization efficiency^22,23^. Nonnenmacher et al.^24^ studied the hollow cone pressure swirl nozzle based on the theory of internal and external flow field of the nozzle, and established the simulation program model of flow coefficient and droplet diameter. According to the simulation program, the Sauter average diameter SMD of the nozzle can be predicted. Other scholars have built deep learning models that can quickly predict spray SMD^25^, Wang et al.^26^ established the pre-film atomization model by using neural network algorithm, and concluded that with the increase of oil pressure difference, the pre-film device appeared anti-fog effect, and the atomization effect was poor. According to the similarity of droplet breakup, Liu et al.^27^ constructed a finite random fragmentation model (FSBM) for the blast atomization process before film formation. The droplet size distribution is simulated by using this model, and the simulation results are consistent with the experimental results of the pre-film blast atomizer. This model can accurately determine the nonlinear relationship between the average droplet diameter SMD and the size distribution of the blast spray. Kaiser et al.^28^ established the suction pressure model of the closed coupled atomization (CCA) nozzle, and applied the machine learning algorithm based on artificial neural network to the prediction of the suction pressure in the tightly coupled HPGA nozzle. The R^2^ of the neural network model is 0.98. According to the parameter research and sensitivity test, the SMD predicted by different working condition parameters can facilitate the conceptual design and operation of the CCA nozzle to minimize the suction pressure. Zhang et al.^29^ used artificial neural network technology to test the prediction accuracy of large eddy simulation (LES) of spray combustion. The results show that the current artificial neural network model can well replicate most species mass fraction tables, which can predict the spray flame of the simulated engine combustion network well.

According to the previous research and analysis of nozzle atomization experiments, there are many studies on the factors affecting the droplet diameter of atomization parameters, and the current nozzle atomization research is limited to the simple relationship and law among atomization parameters, Some people have established the empirical formula atomization model of the traditional nozzle, and the accuracy of the traditional mathematical model is not high, and the error of predicting SMD is large; Few people have established a complex machine learning atomization model for the traditional nozzle atomization parameters, and no one has used supersonic atomization nozzles to establish a machine learning model to predict SMD, and the predecessors have a large prediction error of SMD, and the correlation analysis of SMD influencing factor parameters is more troublesome, we use the neural network algorithm to normalize the data and use the Pearson correlation principle to analyze the speed is faster, time-saving and labor-saving, and the influence weight between each factor can be clearly calculated.

Therefore, in this paper, a new algorithm-BP neural network optimized by genetic algorithm is used to establish an atomization model for the new nozzle atomization parameter droplet average diameter SMD, and compared with the traditional BP neural learning network, a reliable and accurate atomization model is established, which can quickly predict the atomization target parameter SMD, improve the prediction efficiency and accuracy, and adjust the SMD of droplets according to different working conditions. When there is more liquid accumulation at the bottom of the well, the gas flow in the atomization device is less, the liquid flow is more, that is, the gas–liquid ratio is small, the liquid discharge outside the well increases, the drainage efficiency is improved, the liquid accumulation at the bottom of the well decreases, and the natural gas flow channel increases. Drainage and natural gas recovery is greatly improved; when the bottom-hole fluid is less, the natural gas is more, the gas–liquid ratio in the atomizing device is larger, and the recovery rate is improved. The established GA-BP model has certain significance for the setting of downhole nozzle operating parameters and the improvement of drainage efficiency.

## Test method

### Simulation

#### Geometric models

**Figure 1:**
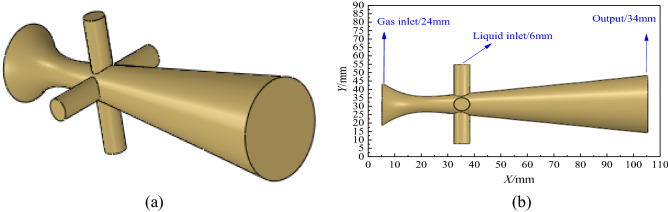
Supersonic atomizing nozzle model. (**a**) Three-dimensional model. (**b**) Model size.

A new type of atomization nozzle based on Laval tube can realize supersonic atomization. The traditional Laval tube is improved and a three-dimensional atomization nozzle model is established as shown in Fig. 1. Figure 1a shows a three-dimensional geometric model of the nozzle, which consists of one gas inlet, four spatially symmetrical liquid inlet and mixing outlet. The downhole natural gas enters from the gas phase inlet of the nozzle and accelerates through the throat, and the flow velocity of the gas reaches supersonic speed. The accumulated fluid enters the cavity from the entrance of the liquid phase and is sheared and broken by the impact of high-speed air flow and atomized. Then it is carried out of the wellbore with the rising natural gas. Figure 1b shows the size of the nozzle model. The gas–liquid inlet diameters are 25.0 mm and 6.0 mm, the mixing outlet diameter is 34.0 mm and the total length is 100.0 mm.

#### Control equations

In this paper, based on the supersonic flow of gas–liquid two-phase flow, the VOF turbulent Realizable k-ε model is used to consider the turbulent eddy current effect. Atomization nozzle makes natural gas produce supersonic flow, high-speed airflow impact liquid flow shear atomization, the continuity equation of mass inflow equal to outflow is:

1 $$\frac{\partial \rho }{\partial t}+\frac{\partial (\rho {u}_{x})}{\partial x}+\frac{\partial (\rho {u}_{y})}{\partial y}+\frac{\partial (\rho {u}_{z})}{\partial z}=0$$

In the formula, ρ is the fluid density, *u*_*x*_*, u*_*y*_ and *u*_*z*_ are the velocity vectors of x, y and z axes respectively.

When the resultant force of the fluid in the cavity is zero, the momentum remains unchanged before and after the momentum. The product of fluid mass and velocity is equal to the time product of the resultant force acting on the fluid, which satisfies the momentum conservation:

2 $$\left\{\begin{array}{c}\frac{\partial (\rho u)}{\partial t}+\nabla \left(\rho {u}_{x}u\right)=div\left(\mu gradu\right)-\frac{\partial p}{\partial x}+{S}_{u}\\ \frac{\partial (\rho u)}{\partial t}+\nabla \left(\rho {u}_{y}u\right)=div\left(\mu gradu\right)-\frac{\partial p}{\partial y}+{S}_{v}\\ \frac{\partial (\rho u)}{\partial t}+\nabla \left(\rho {u}_{z}u\right)=div\left(\mu gradu\right)-\frac{\partial p}{\partial z}+{S}_{w}\end{array}\right.$$

According to the first law of thermodynamics, the internal flow of the cavity satisfies the law of conservation of energy:

3 $$\frac{\partial (\rho E)}{\partial t}+\nabla \cdot \left[u\left(\rho E+p\right)\right]=\nabla \cdot \left({k}_{eff}\Delta T-\sum_{j}{h}_{j}{J}_{j}+{\tau }_{eff}u\right)+S$$

In the formula: *μ* is the dynamic viscosity; *S*_*u*_*, S*_*v*_ and *S*_*w*_ are generalized source terms of momentum conservation equation, *S* is volume heat source term; *p* is the flow field pressure, *E* is the total energy of fluid micelles; *k*_*eff*_ is the effective thermal conductivity; *h*_*j*_ and *J*_*j*_ are the enthalpy and diffusion flux of component *j*; *τ*_*eff*_ is the effective stress tensor; *ΔT* is the fluid temperature gradient.

Realizable *k*-*ε* model:

4 $$\frac{\partial (\rho k)}{\partial t}+\frac{\partial (\rho k{u}_{j})}{\partial {x}_{i}}=\frac{\partial }{\partial {x}_{j}}\left[\left(\mu +\frac{{\mu }_{t}}{{\sigma }_{k}}\right)\frac{\partial k}{\partial {x}_{j}}\right]+{G}_{k}+{G}_{b}-\rho \varepsilon +{S}_{k}$$

5 $$\frac{\partial (\rho \varepsilon )}{\partial t}+\frac{\partial \left(\rho \varepsilon {u}_{j}\right)}{\partial {x}_{j}}=\frac{\partial }{\partial {x}_{j}}\left[\left(\mu +\frac{{\mu }_{t}}{{\sigma }_{\varepsilon }}\right)\frac{\partial \varepsilon }{\partial {x}_{j}}\right]+\rho {C}_{1}{S}_{\varepsilon }-\rho {C}_{2}\frac{{\varepsilon }^{2}}{k+\sqrt{v\varepsilon }}+\frac{{C}_{1\varepsilon }\varepsilon }{k}{G}_{3\varepsilon }{G}_{b}+{S}_{S}$$

Of which: $${C}_{1}=\mathrm{max}\left(0.43\frac{\eta }{\eta +5}\right), \eta =S\frac{k}{\varepsilon }$$

In the equation: *K* is turbulent kinetic energy, *ε* is turbulent dissipation rate; *G*_*k*_ is the turbulent kinetic energy term produced by the laminar velocity gradient; *G*_*b*_ is the turbulent kinetic energy term caused by buoyancy; *Y*_*M*_ is the effect of compressible turbulent expansion on the total dissipation rate; *C*_*1*_*, C*_*2*_ are constants; *σ*_*K*_ and *σ*_*ε*_ are the Prandtl numbers corresponding to k equation and ε equation respectively; *S*_*k*_ and *S*_*ε*_ are user-defined turbulent kinetic energy terms and turbulent dissipation source terms.

#### Boundary conditions and parameter settings

1. Solution method setting
  The VOF gas–liquid two-phase flow model and the turbulence Realizable k-e model are used in the simulation calculation. The implicit solver is selected and the pressure-based solver is used. The transient calculation, the time step is 0.0001 s, the solution time is 50 s, and the coupling of velocity and pressure is SIMPLEC algorithm. The monitor monitor monitors the changes of various physical quantities at the outlet. The residual is set to 10^–6^, and the convergence is more reliable.
2. Boundary condition setting
  The gas inlet is the mass flow inlet, the gas material is air, the initial value is 3000 m^3^/d, converted to a mass flow rate of 42.535 g/s; the liquid nozzle inlet is set as the mass flow inlet, the material is set as liquid water, the initial value is 0.6 m^3^/d, and the mass flow rate is 6.932 g/s. Nozzle outlet end face is set to pressure outlet, the value is an atmospheric pressure − 101,325 Pa, the rest of the wall is set to wall, wall smooth; liquid inlet diameter (*d*), aperture *d* 6.0 mm.

#### Grid independence verification

**Figure 2:**
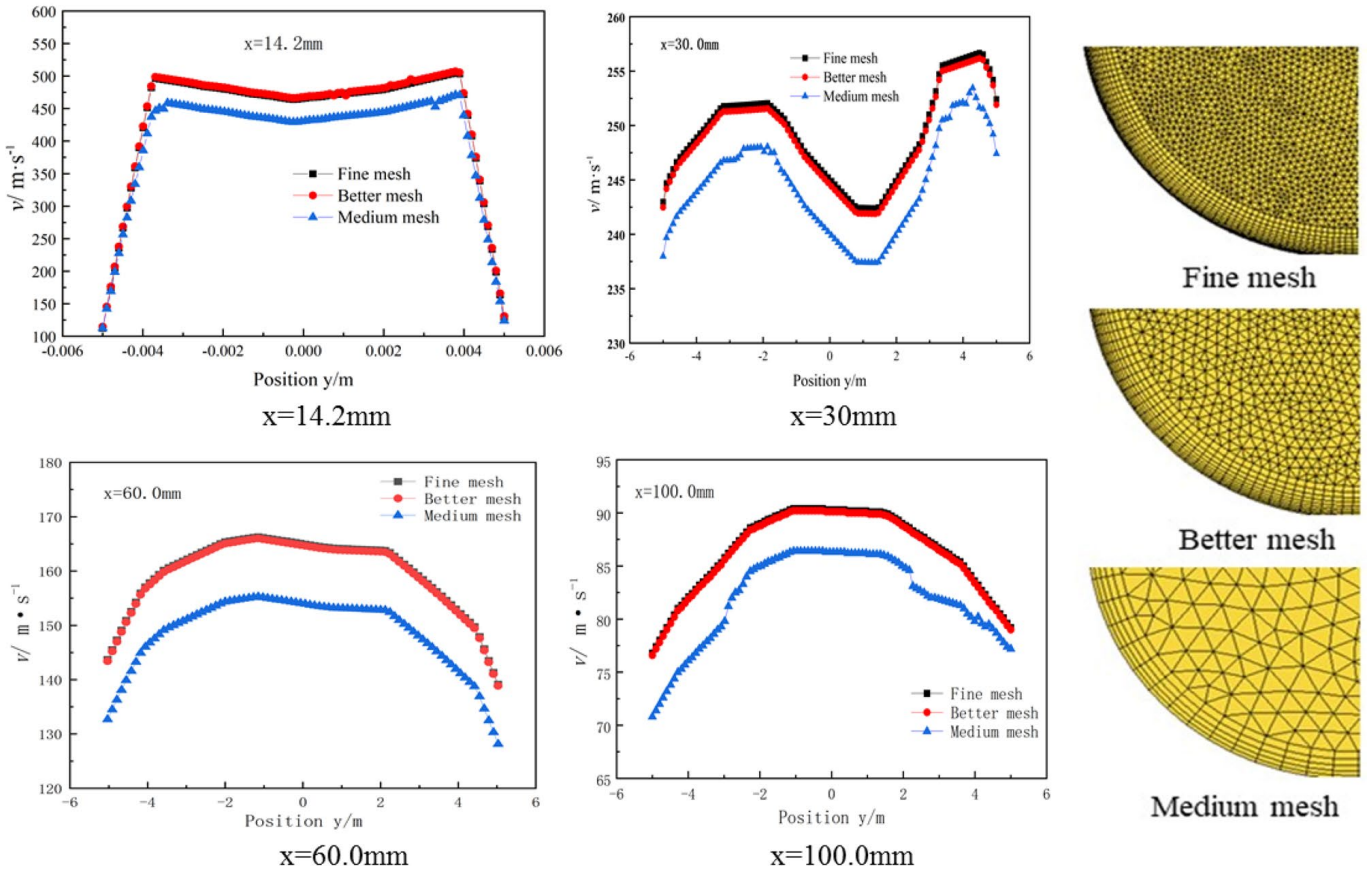
The comparison of the velocity at various cross-sections among three meshing cases.

According to the complex irregular model in this paper, the tetrahedral and boundary layer refinement grid model is adopted, which has high adaptability and grid quality. In general, the more the number of grids, the higher the calculation accuracy, but it will also increase the computational burden. These two factors should be balanced before grid generation. In this paper, three different types (different quantities) of grids are generated, and the independence of the generated grids is tested, as shown in Fig. 2.

During the test, the gas–liquid two-phase velocity of the upstream, middle and downstream four sections of the nozzle x = 14.2 mm, x = 30 mm, x = 60 mm, x = 100 mm were compared. The results show that the velocity simulation results using three different grids have similar trends. The speed of sampling points using better grids is very close to the value using fine grids, and the speed using medium grids is very different from the speed using the other two grids. Therefore, it can be considered that the better grid has met the requirements of grid independence and ensured the simulation accuracy. Using ' better ' grids can effectively shorten the simulation cycle and ensure that the flow field calculation error is small. The final information of mesh ‘better’: the total number of elements is 1,227,661, the total number of nodes is 351,415.

#### Simulation results

**Figure 3:**
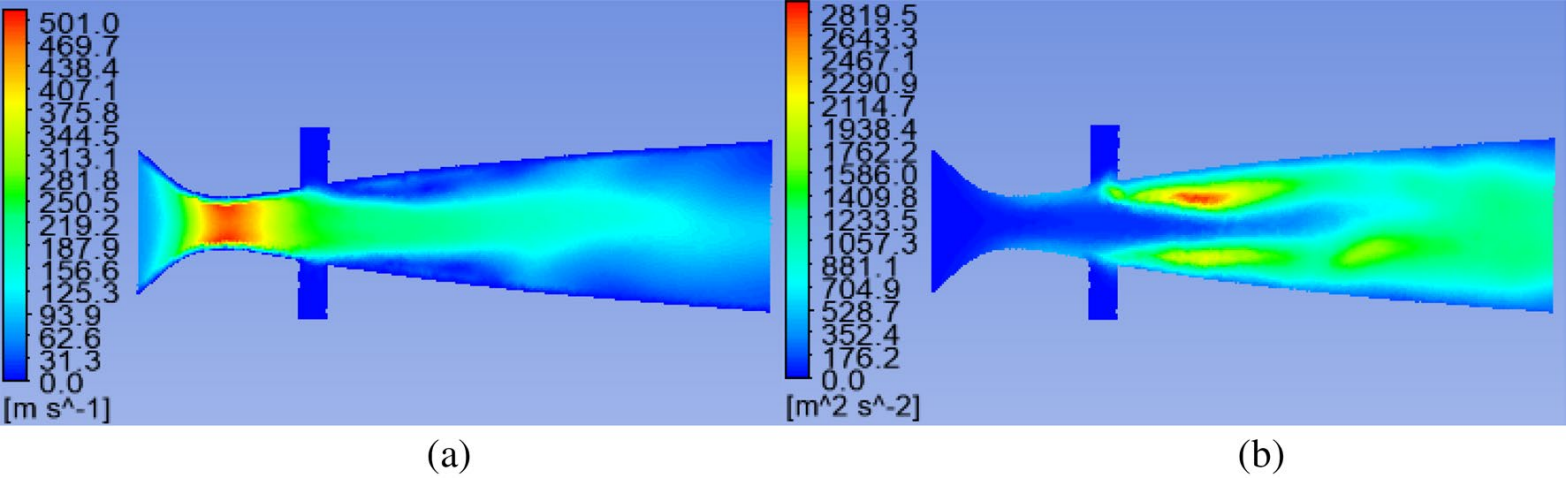
Internal cloud map of supersonic nozzle. (**a**) Velocity cloud map. (**b**) Turbulent kinetic energy cloud map.

Based on Fluent, the simulation is carried out. The initial value of the gas inlet flow is 3000 m^3^/d, the initial value of the liquid inlet flow is 0.6 m^3^/d, the initial value of the liquid inlet diameter is 6.0 mm, and the outlet pressure is an atmospheric pressure of 101325 Pa. The calculated internal cloud diagram of the nozzle is shown in Fig. 3. The maximum velocity of the throat is 501.0 m/s to reach supersonic speed, and the maximum turbulent kinetic energy is 2819.5 m^2^/s^2^.

**Figure 4:**
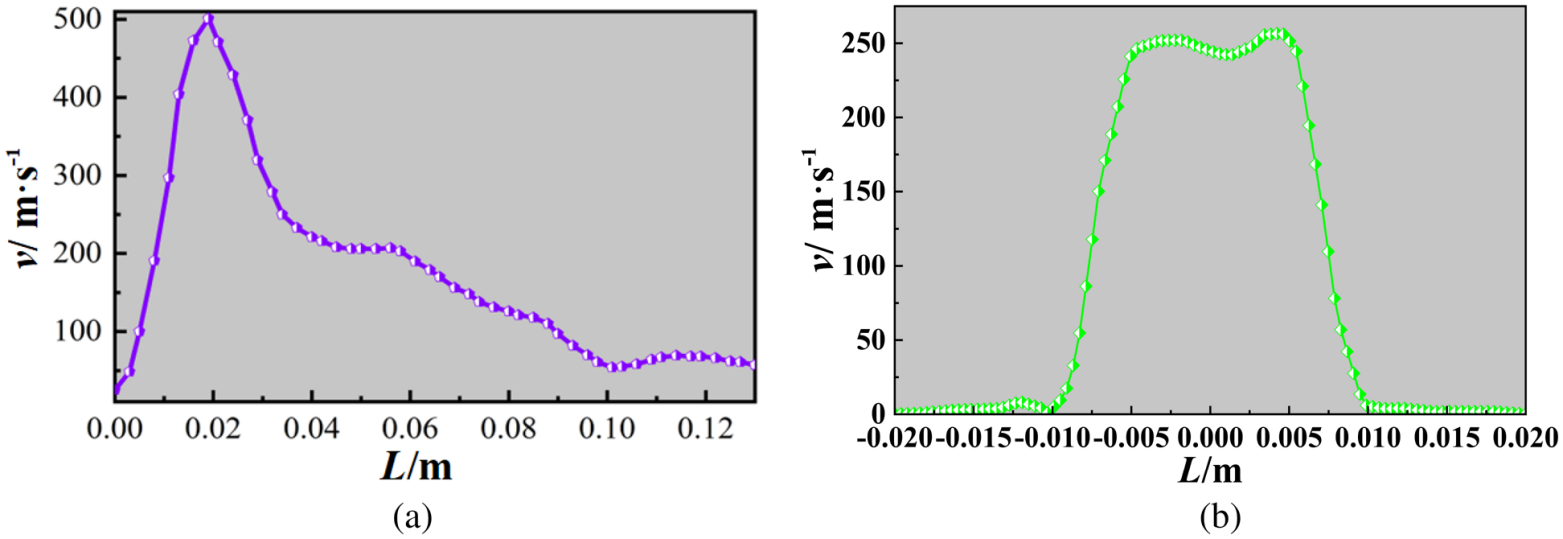
Internal cloud map of supersonic nozzle. (**a**) Transverse central axis. (**b**) Longitudinal axis velocity.

According to the internal flow field of the atomizing nozzle, a transverse central axis is established inside the atomizing nozzle. The transverse velocity distribution inside the nozzle is shown in the figure. When the throat velocity reaches a maximum of 501.0 m/s, it is 18.97 mm from the gas inlet. Similarly, the longitudinal axis data of the liquid inlet are shown in the Fig. 4. The calculated axial velocity at the liquid inlet of the liquid flow is 0.24 m/s, and the maximum longitudinal velocity is 256.73 m/s. It collides with the supersonic airflow and shears. The turbulence is severe, the gas–liquid phase interaction improves the atomization effect.

### Experimental study

#### Experimental system

**Figure 5:**
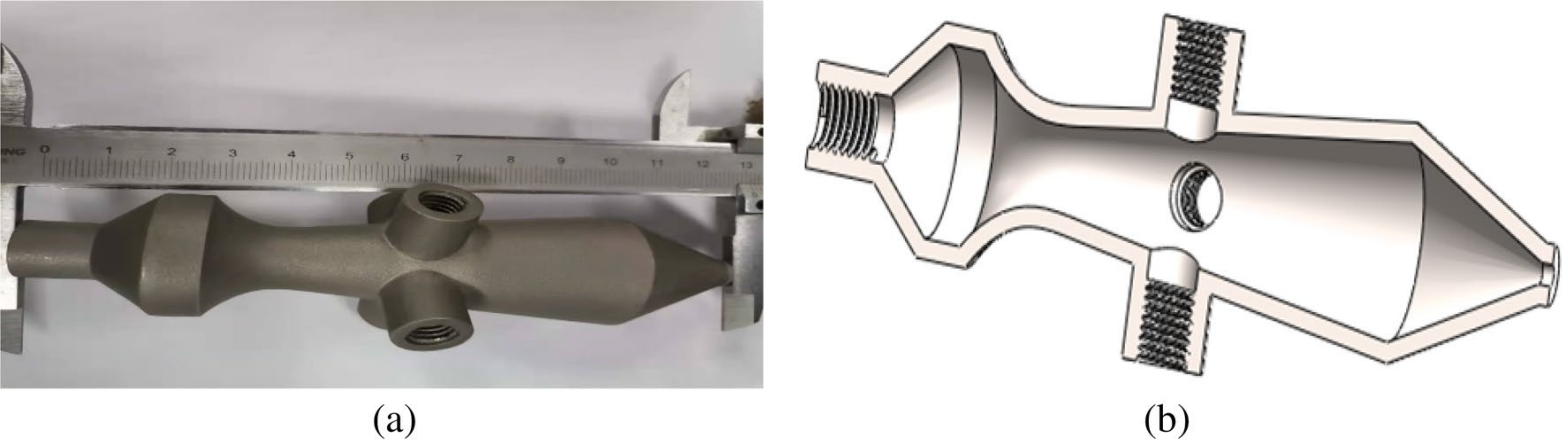
Working atomizing nozzle model. (**a**) Nozzle physical object. (**b**) Internal cross section of nozzle.

According to the simulated supersonic nozzle model, the supersonic atomization working nozzle is improved and established by Solidworks software, and then the porous symmetrical working atomizer is made by 3D printing technology, as shown in Fig. 5. Figure 5a shows the working atomizer, made of stainless steel, with a total length of 130 mm. Figure 5b shows the internal profile of the working atomizer.

**Figure 6:**
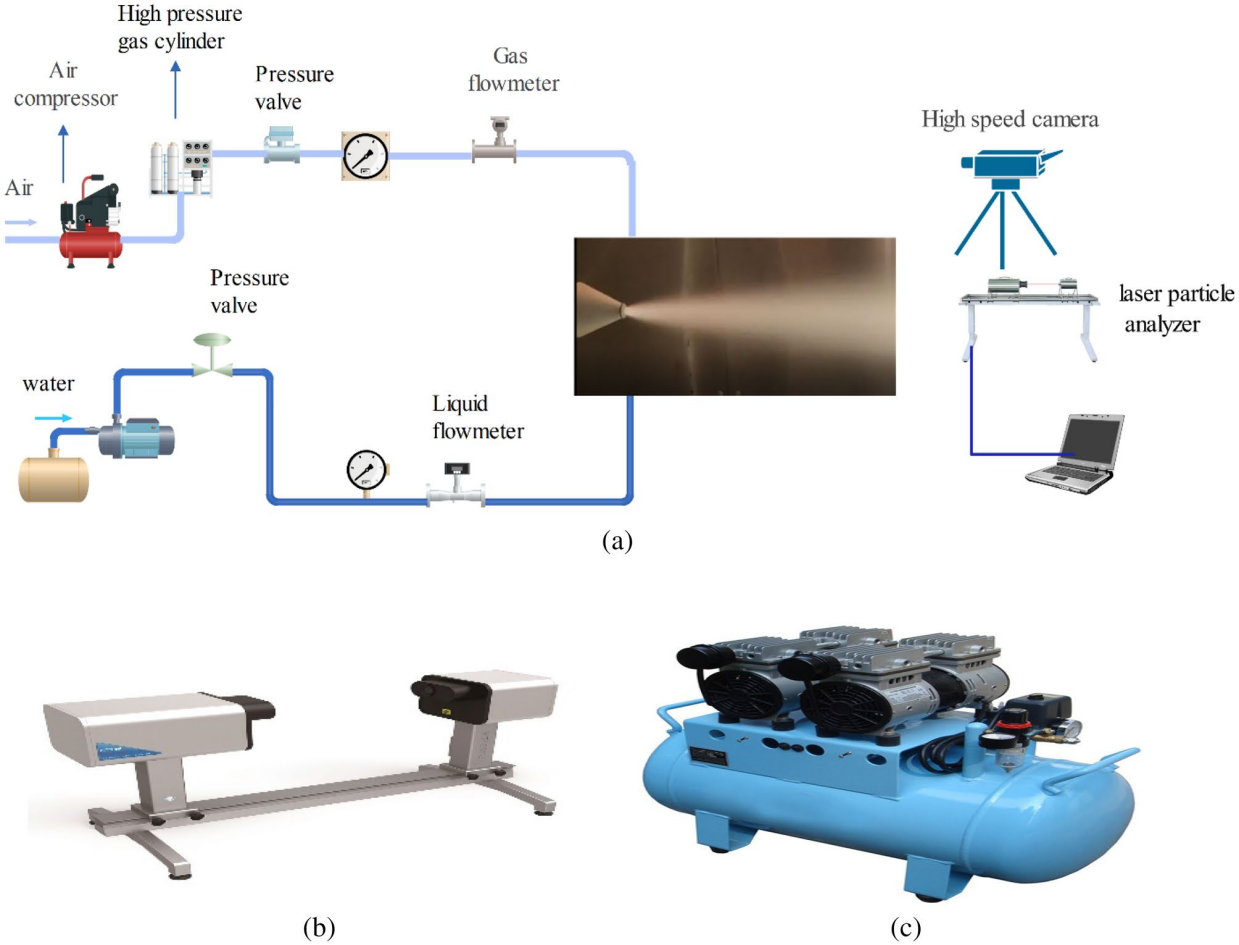
Atomization experiment work platform. (**a**) Experiment platform. (**b**) Laser particle size analyzer. (**c**) Air compressor.

A spray experimental system is established as shown in Fig. 6. Figure 6a shows a manual experimental platform, including high-pressure water pump, liquid Flowmeter, air compressor, high-pressure gas cylinder, gas Flowmeter, high-speed camera, laser particle size analyzer, computer and so on.

Among them, the gas flowmeter model is HL-LWQ, measuring range is 1–10,000 (m^3^/h), working temperature 0–60 °C; the model of liquid flowmeter is LWGY-FMT, measuring range is 0.6–800 (m^3^/h), working temperature is − 20 to 120 (°C), pressure gauge model is YTZ-150, measuring range is − 0.1 to 0–60 (MPa), working temperature is − 10 to 80 (°C).

Figure 6b shows a laser particle size analyzer. The droplet size was measured by HELOS/VARIO-KR laser particle size analyzer in Germany. The laser particle size meter has a wide range of droplet size measurement, and can cover the parallel optical path design of 0.1–3500 μm dynamic range according to the selection of different lenses, and can realize wide spray plume particle size measurement.

The particle size analyzer is connected with the computer, and the data is processed by the computer. The droplet diameter measured by the laser particle size analyzer was statistically analyzed by using Sotel's average particle size SMD. SMD is represented by *D*_*32*_, and the calculation equation is:

6 $$ {\text{D}}_{32} = \frac{{\smallint_{\min }^{\max } D^{3} dN}}{{\smallint_{\min }^{\max } D^{2} dN}} $$

where, *D*_*32*_ is the average particle size of Sauter, and N is the number of droplets with diameter D.

Figure 6c shows KJ series industrial air compressors. The air compressor has a discharge capacity of 0.36–0.9 m^3^/min, maximum exhaust pressure 2.5 Mpa, maximum motor power 7.5 KW, safe and reliable, and long service life.

#### Experimental scheme

In order to simulate the gas–liquid atomization flow state of low-pressure and low-yield gas wells, the gas volume flow (*Q*_*g*_) is 3000 m^3^/d, 3500 m^3^/d, 4000 m^3^/d, 4500 m^3^/d and 5000 m^3^/d, and the liquid volume flow (*Q*_*l*_) is 0.6 m^3^/d, 1.0 m^3^/d, 1.4 m^3^/d, 1.8 m^3^/d, and 2.2 m^3^/d. The diameter of the liquid phase nozzle d is 6.0 mm, 6.6 mm, 7.2 mm, 7.8 and 8.4 mm. The orthogonal test of 3 factors and 5 levels was designed. The level factors are shown in Table 1.

**Table 1:** Test factor level table.

Factor	*Q*_*g*_-(m^3^/d)	*Q*_*l*_-(m^3^/d)	*d*-mm
Level 1	3000	0.6	6.0
Level 2	3500	1.0	6.6
Level 3	4000	1.4	7.2
Level 4	4500	1.8	7.8
Level 5	5000	2.2	8.4

The 3-factor 5-level orthogonal experimental model was set to 60 groups, and the experimental orthogonal table combined the levels of each influencing factor with equal probability, the sequence of orthogonal experiment is shown in Table 2.

**Table 2:** Orthogonal experiment sequence table.

Experimental sample	*Q*_*g*_/m^3^/d	*Q*_*l*_/m^3^/d	*d*/mm
1	3000	0.6	6.0
2	3000	1.0	6.6
3	3000	1.4	7.2
4	3000	1.8	7.8
5	3000	2.2	8.4
	…	…	…
57	5000	1.8	7.8
58	5000	2.2	8.4
59	5000	1.0	7.2
60	5000	1.4	7.8

The experiment is carried out indoors, the indoor environment is stable, the temperature is 25 °C at room temperature, the working principle of the experimental system is: the high-pressure air flow enters the contraction section of the atomization nozzle from the gas phase inlet through the gas flow meter, the water flows through the liquid phase inlet into the expansion section, and the high-speed air flow accelerated to the expansion section by the throat collides with the water flow and shears atomization and sprays out through the mixing outlet; At the same time, the spray flow field is ingested by a high-speed camera, and the particle size distribution of the droplets is measured by the laser particle size meter at the monitoring point at the uniform atomization place, and the data of the atomized droplet diameter under different working condition parameters is collected. After the experiment, first turn off the power, then turn off the air compressor and water pump, then close the pressure valve, and finally perform data output preprocessing.

The specific operation of the test is as follows:

1. First, connect each experimental equipment in order, place the laser particle size detector at the exit of the atomization nozzle, connect it to the computer, and check whether the safety and stability of the system are normal;
2. Secondly, open the switch of the pressure control valve and pressure gauge, start the laser particle size tester, and then turn on the air compressor and other nozzles after stabilizing and then open the water pump, in which the air is compressed and stored in the high-pressure gas cylinder, and the parameters required by the air compressor and water pump experiment are set;
3. The laser particle size tester is placed at 50 cm of the nozzle outlet, and the atomization droplet data collection and data output are carried out after the atomization nozzle spray is stabilized;
4. Record the changes of the pressure gauge flowmeter in real time, monitor and record the pressure gauge and flowmeter;
5. After the experiment is completed, first turn off the power supply, then turn off the air compressor and water pump, then close the pressure valve, and finally carry out data output preprocessing.

### Atomization model establishment

#### BP neural network

**Figure 7:**
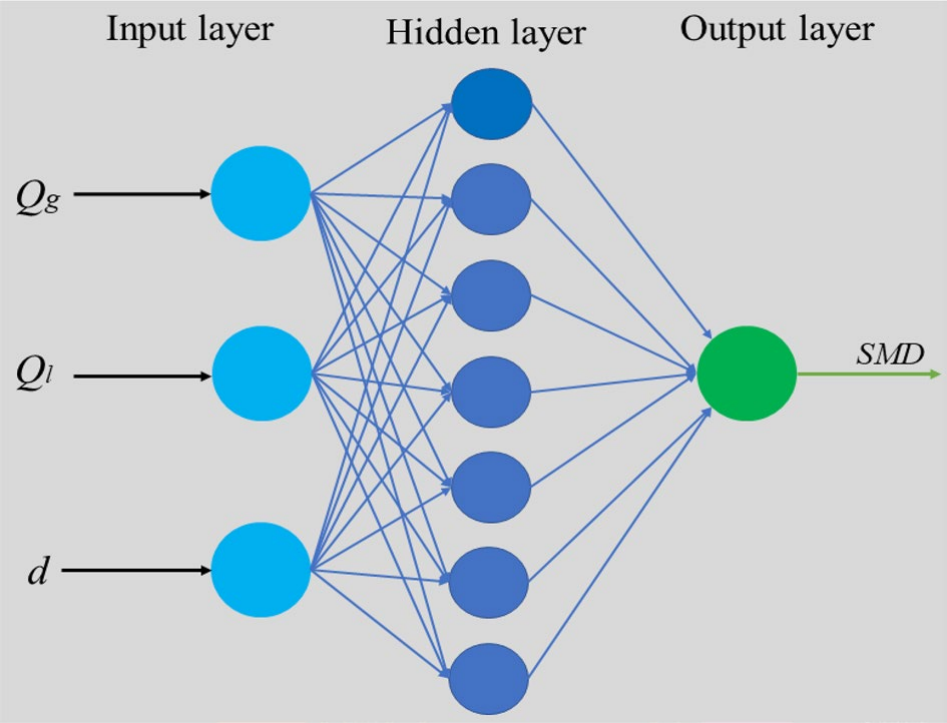
Schematic diagram of BP neural network.

Neural network prediction is widely used in production practice, has strong learning and adaptive ability, and can effectively connect input and output information in series. BP neural network is a "universal model + error correction function". It is a model which can compare the error between the training results and the expected results, modify and find the optimal weight and threshold, and gradually get the model which is consistent with the expected output results. BP neural network consists of input layer, hidden layer and output layer. The diagram of the network structure is shown in Fig. 7.

Each neuron is stimulated by other neurons. All the signals received by each neuron model are transmitted through a weighted connection. The neuron accumulates these signals to a total input value, and then compares the total input value with the threshold value of the neuron (simulated threshold potential), which is processed by the "activation function" and transmitted to the next neuron. The activation function is a nonlinear mapping function. The effect of activation function is to increase the nonlinear ability of the model, make it form nonlinear mapping, and enhance the adaptability and reliability of the model. The activation function applies to the weighted sum of inputs called z for each node in the hidden and output layers (where the input can be either the original data or the output from the previous layer). The sigmoid function converts its input to a probability value between 0 and 1. It converts large negative values to 0 and large positive values to 1.

The activation function equation is:

7 $$ {\text{sigmoid}} (z) = \frac{1}{{1 + e^{ - z} }} $$

where, the number of nodes in the hidden layer of BP network affects the accuracy of model training. The more the number of nodes, the more reliable the training quality. Hidden nodes can be calculated according to empirical equation:

8 $$ m = \sqrt {n + l} + a $$

where, n is the number of nodes in the input layer; m is the number of hidden layer nodes; *l* is the number of output layer nodes; a is a constant between 0 and 10.

The input node and target output parameters of BP neural network are shown in Table 1. The input nodes are gas flow rate, liquid phase flow rate and liquid phase inlet diameter, and the target output layer is droplet diameter SMD.

#### Genetic algorithm

Genetic algorithm is a computational model that simulates the biological evolution process of Darwin's biological evolution theory, and it is a method to search the optimal solution by simulating the natural evolution process. Genetic algorithms start by representing the potential solution set of the problem, while a population is made up of a certain number of individuals encoded by genes. At the beginning, many feasible solutions are randomly selected (to form a population), each individual is composed of a chromosome containing only two genes (0 or 1), and the chromosomes of different individuals are different. but they can be crossed with each other, or mutated and crossed, and then the initial parameters are evaluated and screened.

1. Population initialization
  The initial population is randomly generated by the data parameters, and the population size directly affects the chromosome diversity. Chromosomes are made up of arrays and data strings. Initialization randomly generates N initial string structure data, and N individuals form a group. GA began to evolve with these N string structure data as the starting point.
2. Fitness function
  Fitness function is also called evaluation function. The effect of genetic algorithm evaluation of algorithm convergence depends on the fitness value, not on the structure of the solution. The size of the fitness value determines the advantages and disadvantages of the individual. The larger the fitness value is, the better the individual is. Therefore, our criteria for selecting individuals are selected by the size of the fitness value. The fitness function is used to calculate the fitness value of each individual, and the results are provided to the selection operator.
  9 $$ F_{i} = k\left( {\sum\limits_{i = 1}^{n} {\text{abs}} \left( {y_{i} - x_{i} } \right)} \right) $$
  where, n is the number of output nodes of the network, y_i_ is the expected value of the i node of the BP neural network, x_i_ is the actual output of the i node, and k is the coefficient.
3. Selection operation
  The selection operation is to select relatively excellent individuals in the population to prepare for the next operation. When the roulette method is used in the selection operation of genetic algorithm, it is a selection strategy based on fitness ratio to select some data with high fitness value. The higher the adaptive performance, the higher the output precision. The probability of an individual being selected, *p*_*i*_, is:
  10 $$ f_{i} = \frac{k}{{F_{i} }} $$
  11 $$ p_{i} = \frac{{f_{i} }}{{\sum\nolimits_{j = 1}^{N} {f_{j} } }} $$
  where, F_i_ is the fitness value of i individual, and the reciprocal of fitness degree before individual selection is *f*_*i*_; k is the coefficient.
4. Crossover operation
  Crossover operation refers to the selection of two individuals from all individuals, by using the cross-combination of two chromosomes to check the excellent individuals. In the crossover process, two chromosomes are randomly selected from the population, and one or more unknown chromosomes are randomly selected for exchange. Because the individual uses real number coding, the real number crossing method is used in the crossover operation method. The crossover operation of the k-th chromosome ${a}_{kj}$ and the i-th chromosome ${a}_{ij}$ at position j is as follows:
  12 $$ \left\{ {\begin{array}{*{20}l} {a_{kj} = a_{kj} (1 - b) + a_{lj} b} \hfill \\ {a_{lj} = a_{lj} (1 - b) + a_{kj} b} \hfill \\ \end{array} } \right. $$
  where, b is a random number between [0,1].
5. Mutation operation
  Mutation operation means to select an individual from the population, select a point of the chromosome to mutate to produce a better individual, and mutate the individual gene of the population. The mutation operation function using the j-th gene ${a}_{ij}$ of the i individual is as follows:
  13 $$ a_{ij} = \left\{ {\begin{array}{*{20}l} {a_{ij} + \left( {a_{ij} - a_{\max } } \right)*f(g)} \hfill & {r > 0.5} \hfill \\ {a_{ij} + \left( {a_{\min } - a_{ij} } \right)*f(g)} \hfill & {r < = 0.5} \hfill \\ \end{array} } \right. $$
  14 $$ f(g) = r_{2} \left( {1 - \frac{g}{{G_{\max } }}} \right)^{2} r_{2} $$
  where, ${a}_{\mathrm{max}}$ is the upper bound of gene ${a}_{ij}$, ${a}_{\mathrm{min}}$ is the lower bound of gene ${a}_{ij}$, g is the current evolutionary algebra, *G*_*max*_ is the maximum evolution value of 20, *r* is the random number of [0,1].

#### GA-BP neural network

**Figure 8:**
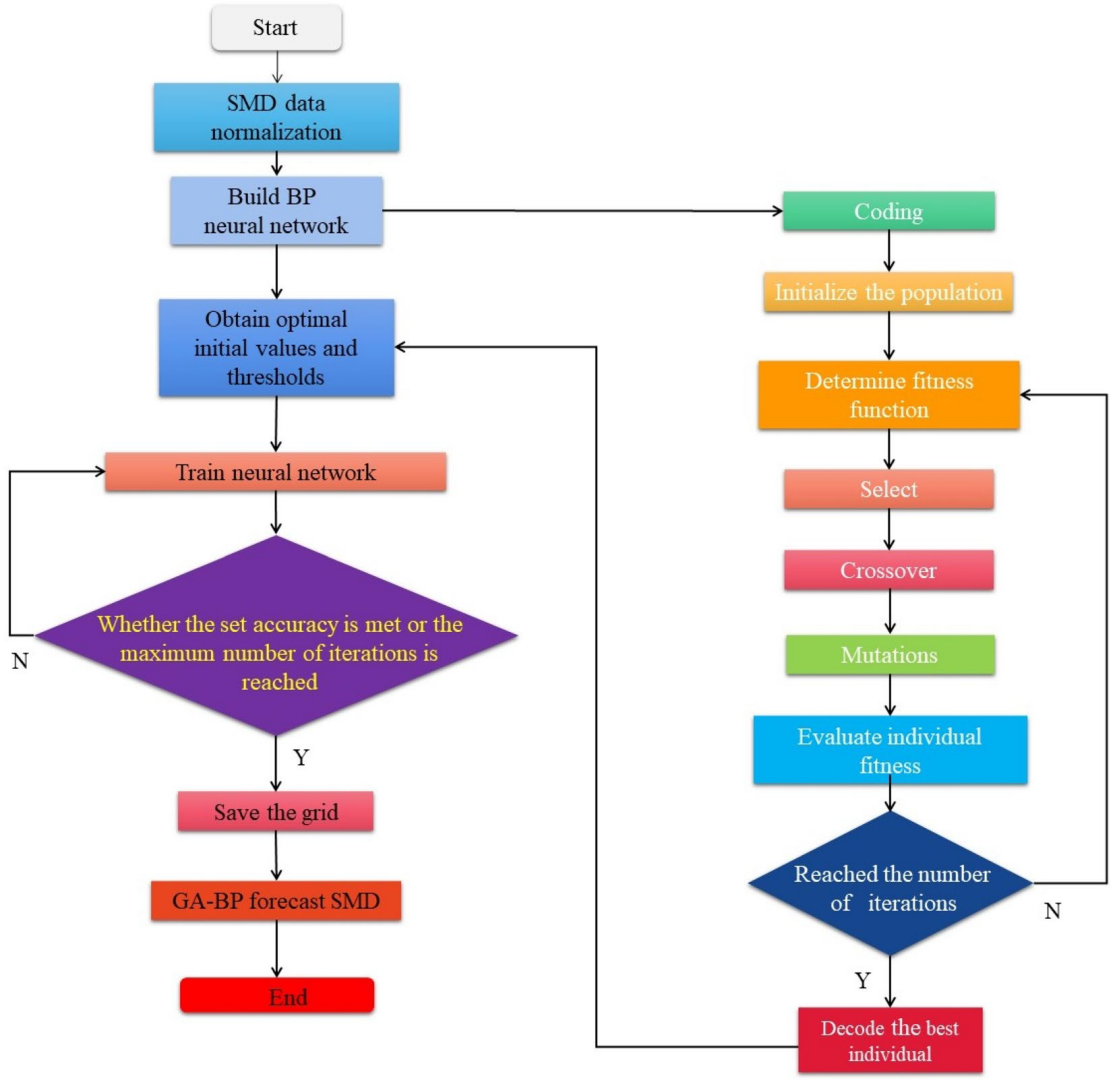
The processing flow of GA-BP neural network.

BP algorithm has a good effect on the fitting of nonlinear systems, but it has some limitations. The weights and thresholds of network training are unstable, and the effect of each training is not fixed. Although it has a strong ability of nonlinear mapping, it takes more time in the process of approaching the predicted value, which leads to slow convergence and easy to fall into local optimization. In the study of practical problems, genetic algorithm has better global search ability and can obtain the global optimal solution with fast convergence speed. In order to improve the training effect of the network, genetic algorithm is introduced to reduce the possibility that the BP neural network is easy to fall into local optimization. BP neural network optimized by genetic algorithm can preliminarily screen the random weights of neural network and optimize the network structure, which greatly improves the learning and adaptive performance of BP neural network, and can achieve the best prediction effect. The flow of BP neural network optimized by genetic algorithm is shown in Fig. 8.

### Model evaluation

In this paper, mean square error (MSE), average absolute error (MAE) and average absolute percentage error (MAPE)are used to evaluate the performance of the prediction model, and the quality of model prediction is determined by these three indexes. The smaller the MSE is, the closer the MAE and MAPE are to 0, indicating that the relative error of the model prediction is smaller and the overall prediction accuracy is higher.

15 $$ MSE = \frac{1}{n}\sum\limits_{i = 1}^{n} {\left( {y_{i} - x_{i} } \right)^{2} } $$

16 $$ MAE = \frac{1}{n}\sum\limits_{i = 1}^{n} {\left| {y_{i} - x_{i} } \right|} $$

17 $$ MAPE = \frac{1}{n}\sum\limits_{i = 1}^{n} {\left| {\frac{{y_{i} - x_{i} }}{{y_{i} }}} \right|} $$

where, *x*_*i*_ is the i predicted value; *y*_*i*_ is the *i* actual value。

The determination coefficient R^2^ was used to evaluate the fitting degree between the predicted experimental values of SMD and the simulated values. The closer R^2^ is to 1, the better the model is. The equation for R^2^ is:

18 $$ R^{2} = 1 - \frac{{\sum\nolimits_{i = 1}^{n} {\left( {x_{exp} - x_{{{\text{pred}}}} } \right)^{2} } }}{{\sum\nolimits_{i = 1}^{n} {\left( {x_{\exp } - \overline{x}_{e \times p} } \right)^{2} } }} $$

where, *x*_exp_ is the experimental value, *x*_pred_ is the predicted value,$\bar{X}_{\text{exp}}$_exp_ is the average value of the experimental value.

### Data normalization

Because of the large dimension gap between different dimensions in the original data, it is necessary to normalize the data in order to make different types of data at the same latitude and interval. This can effectively reduce data differences, improve adaptability, reduce data redundancy, and improve data parameter operation accuracy and convergence efficiency. In this paper, 60 groups of experimental values are selected as the sample set, the data are normalized, and mapped to the range of [0,1]. Use the Premnmx function to normalize the original data as follows:

19 $$ x^{\prime } = \frac{{x - x_{\min } }}{{x_{\max } - x_{\min } }} $$

The formula *x*_*max*_ and *x*_*min*_ are the maximum and minimum values of SMD for what experimental samples.

## Test results and analysis

### Simulation test parameters

The input parameters of BP neural network are gas flow rate, liquid phase flow rate, liquid phase inlet diameter and average droplet size SMD. In the experimental study, 60 groups of parameters were trained and tested, as shown in Table 3. All the experimental data are attached.

**Table 3:** Experimental parameters and results.

*Q*_*g*_/m^3^/d	*Q*_*l*_/m^3^/d	*d*/mm	*SMD*/μm
3000	0.6	6.0	148.00
3000	1.0	6.6	164.91
3000	1.4	7.2	177.89
3000	1.8	7.8	188.66
3000	2.2	8.4	198.27
…	…	…	…
5000	1.8	7.8	74.45
5000	2.2	8.4	84.06
5000	1.0	7.2	56.65
5000	1.4	7.8	70.15

The GA-BP network model first normalizes the experimental data, and then debugs the simulation program. The initial parameters of the selected model are shown in Table 4:

**Table 4:** GA-BP neural network simulation initial parameters.

Data name	Data parameter
Population size	20
Learning rate	0.05
Crossover probability	0.2
Mutation probability	0.1
Number of training	1000
Maximum evolution algebra	20
Parameter dimension	4
BP network structure	3-7-1

### Model training

In this paper, according to BP neural network and BP network optimized by genetic algorithm (GA-BP), the training set can be accurately predicted and tested by sample data. Here, the first 45 sets of experimental data sets are used to learn and train the atomization model.

**Figure 9:**
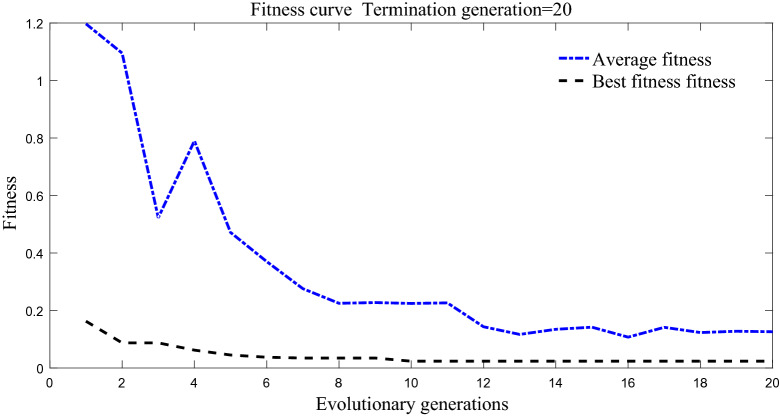
GA-BP fitness response curve.

Genetic algorithm is used to optimize the structure of BP neural network 3-7-1 in simulation. Genetic algorithm can transfer the optimal threshold and weight to BP neural network. The changes of the best fitness and the average fitness of the population are shown in Fig. 9. In the initial stage of iteration, the individual of the population is far from the expected value. In the later stage of iterative optimization, with the continuous iterative convergence of network calculation, the search speed of the optimized genetic algorithm increases, the range of fitness value decreases, and the decline speed is accelerated. The average fitness of the population decreases with the evolutionary algebra, and the individual of the population gradually approaches the optimal fitness.

**Figure 10:**
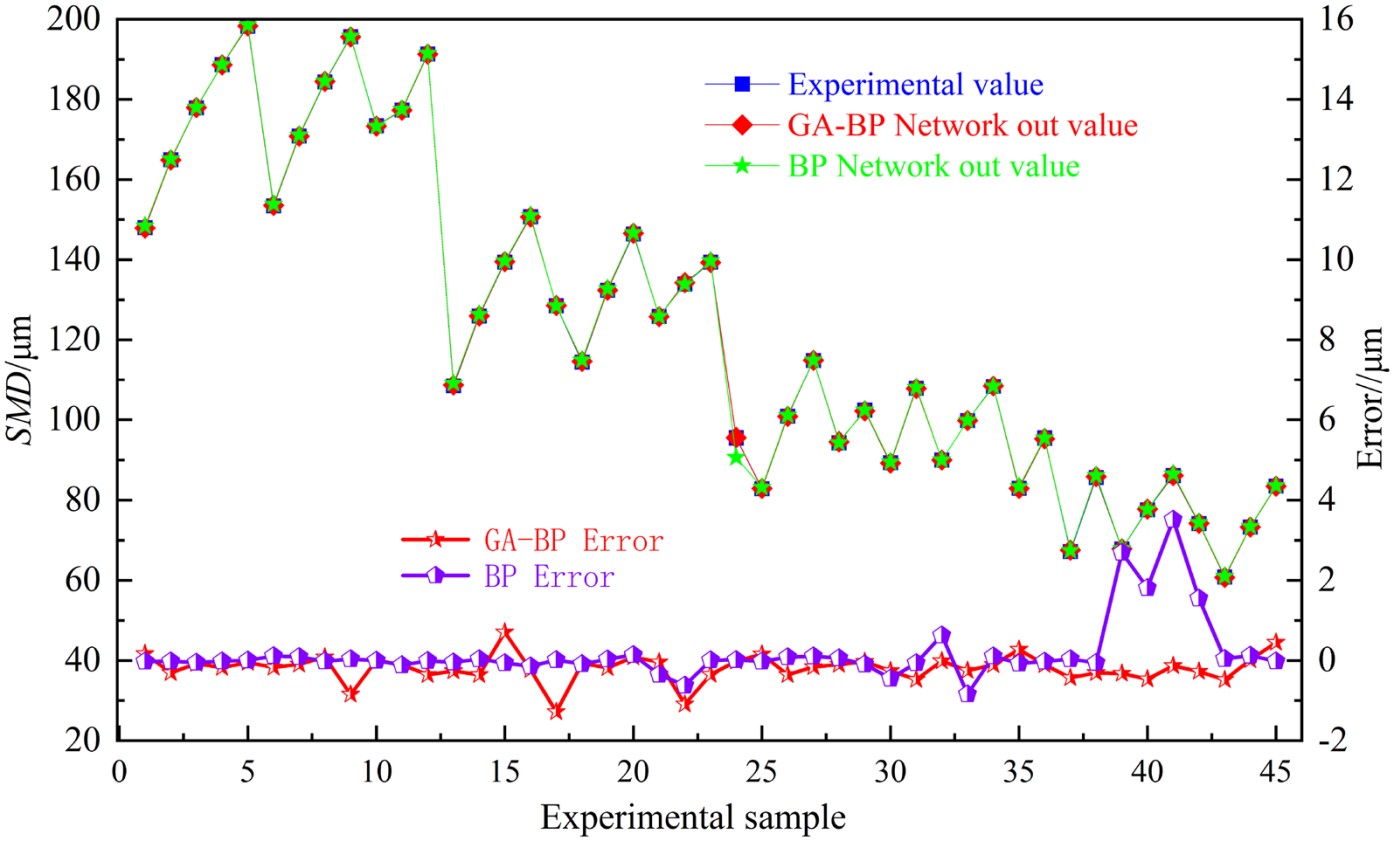
Model training error.

The BP network is trained by using the weight threshold optimized by GA. The training results of the learning model are shown in Fig. 10. The training effect of SMD atomization model is good. The SMD training values of GA-BP and BP are in good agreement with the experimental values. The training error of GA-BP is smaller than that of BP network, because the BP network optimized by genetic algorithm has stronger adaptability and more stable network structure.

### Sensitivity analysis

**Figure 11:**
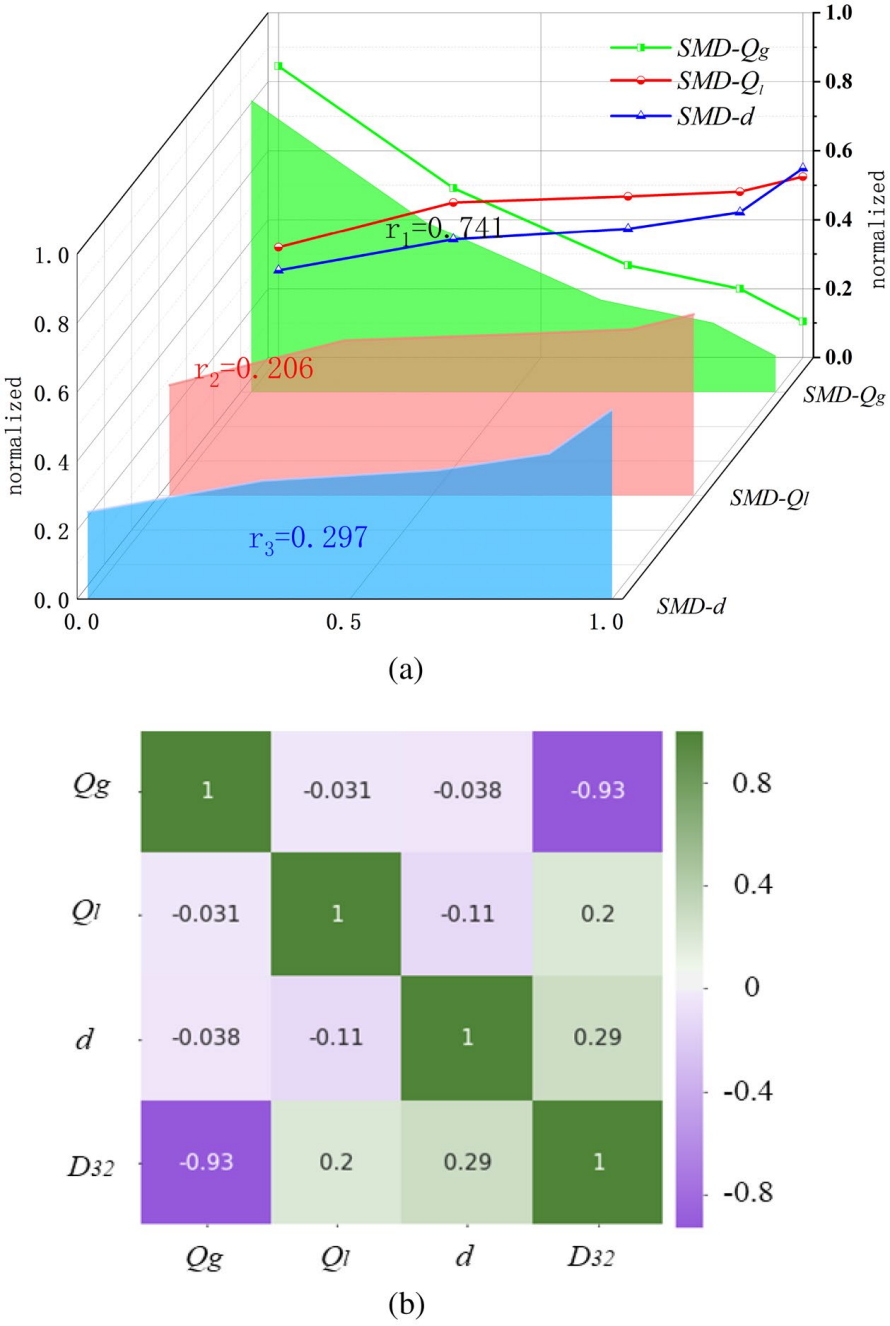
Sensitivity of each input parameter to the normalized SMD of the BP network. (**a**) Standard normalization. (**b**) Correlation coefficient matrix plot.

According to the atomization model trained by BP network, the gas flow rate, liquid phase flow rate and liquid phase inlet diameter are input variables, and droplet size is output variables. The relationship between the normalized SMD and the normalized input parameters is shown in Fig. 11.

In Fig. 11a, the normalized SMD decreases with the increase of the supply flow rate Qg. Because of the increase of air supply, the kinetic energy in the nozzle cavity increases rapidly, the shear crushing effect is strengthened, and the atomization performance is better; SMD tends to increase with the increase of liquid supply. Because the total number of droplets increases, the total volume of droplets increases, the effective surface area of droplets decreases, and the average particle size of droplets increases; With the increase of liquid inlet diameter, SMD increases the liquid flow rate and the average droplet size.

Figure 11b shows the Pearson correlation coefficient thermal map generated by the normalized parameters of the BP network. There is a negative correlation between *Q*_*g*_ and SMD, and the sensitivity is the strongest, and the correlation is − 0.93, which is consistent with the change trend of *Q*_*g*_ and SMD in Fig. 10a. There is a positive correlation between *Q*_*l*_, d and SMD, and the correlation values are 0.20, 0.29, respectively. The *d* has a stronger effect on SMD than *Q*_*l*_. To sum up, the relationship of sensitivity to SMD is Q*g* > *d* > *Q*_*l*_.

### Model testing

**Figure 12:**
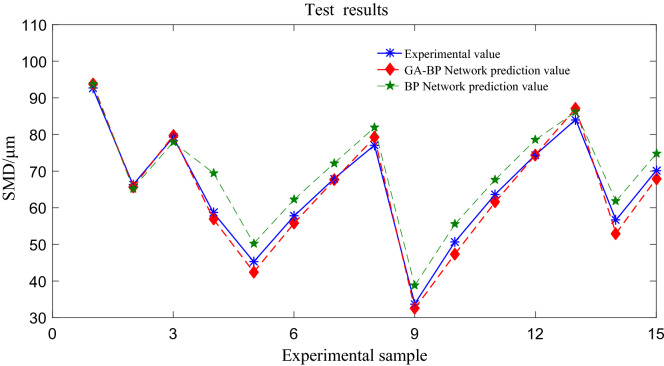
GA-BP test results.

The model training is more accurate. The model is tested by using the data outside the training sample, and the atomization model is tested by the test set data. When the network training results meet certain accuracy requirements, the remaining 15 groups of data are used to test the GA-BP network. The predicted results of the model are shown in Fig. 12, the specific experimental prediction data are shown in Table 5. The prediction error of BP network is larger than that of GA-BP network, and the prediction error of GA-BP network is smaller. The predicted values are in good agreement with the experimental values.

**Table 5:** GA-BP neural network predicts droplet size SMD results.

Experimental value	BP predicted value	BP error	GA-BP predicted value	GA-BP error
92.64	93.74	1.104	93.86	1.224
66.25	65.27	− 0.981	65.64	− 0.614
79.23	77.88	− 1.355	79.71	0.475
58.63	69.49	10.857	56.86	− 1.776
45.25	50.27	5.013	42.43	− 2.821
57.72	62.20	4.479	55.72	− 1.997
67.98	72.18	4.198	67.57	− 0.409
77.08	81.90	4.822	79.18	2.093
33.78	38.76	4.977	32.48	− 1.299
50.70	55.52	4.821	47.36	− 3.339
63.68	67.69	4.015	61.55	− 2.124
74.45	78.54	4.089	74.44	− 0.003
84.06	86.20	2.141	87.16	3.094
56.65	61.79	5.133	53.01	− 3.657
70.15	74.74	4.595	67.91	− 2.234

The prediction result of GA-BP network is closer to the real SMD value than that of unoptimized BP network, and the prediction accuracy of GA-BP is better than that of BP, and the prediction accuracy of BP network needs to be improved. This is because: 1. The sample data set is not infinite, and the network does not fully study the fluctuation trend of SMD. 2. The average droplet size is not only related to the experimental input variables, but also related to the changing factors of the laboratory environment, such as temperature, humidity, noise and so on, so the output signal distortion may occur in BP network prediction. While GA-BP selects and optimizes the random values of the network through selection, mutation and crossover, and the prediction output accuracy is greatly improved.

### Error analysis

**Figure 13:**
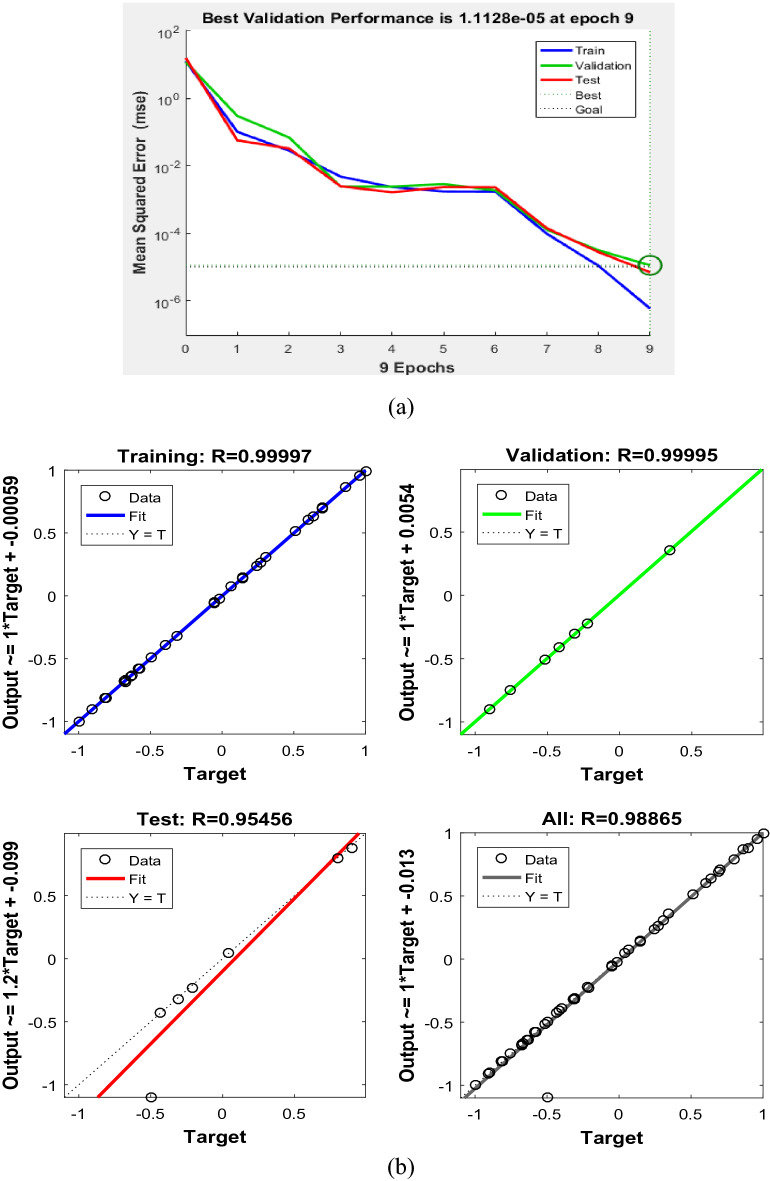
Training results. (**a**) Model training MSE. (**b**) Correlation coefficient of GA-BP model.

Figure 13A shows the average error response curve of GA-BP network training. The experimental results show that the BP network improved by genetic algorithm can adaptively adjust the crossover and mutation rate of individuals in the number of populations, accelerate its convergence speed, and effectively reduce the number of training steps. The optimal evolution algebra is the 9th generation, and the minimum convergence value of MSE is 1.1128 × 10^–5^.

Figure 13b is a matrix data graph with 2 rows and 2 columns. The abscissa is the target value, the ordinate is the output value of the network, and the diagonal line (-Fit) is the best adaptive fitting curve of the target value and the network output value. In order to prevent over-fitting, the method used by MATLAB is to divide the data into three parts. Training is the training data, validation and test are the validation data and test data respectively. The correlation between the trained network output value and the training target value is 0.99997, (b upper left). As the training progresses, the error between the training output data and the training target data becomes smaller and smaller, and the validation and test data are the same. The error is getting smaller and smaller. The correlation between all network output values and all target values in the fourth figure (lower right) is 0.98865, and finally a perfect regression is formed.

**Figure 14:**
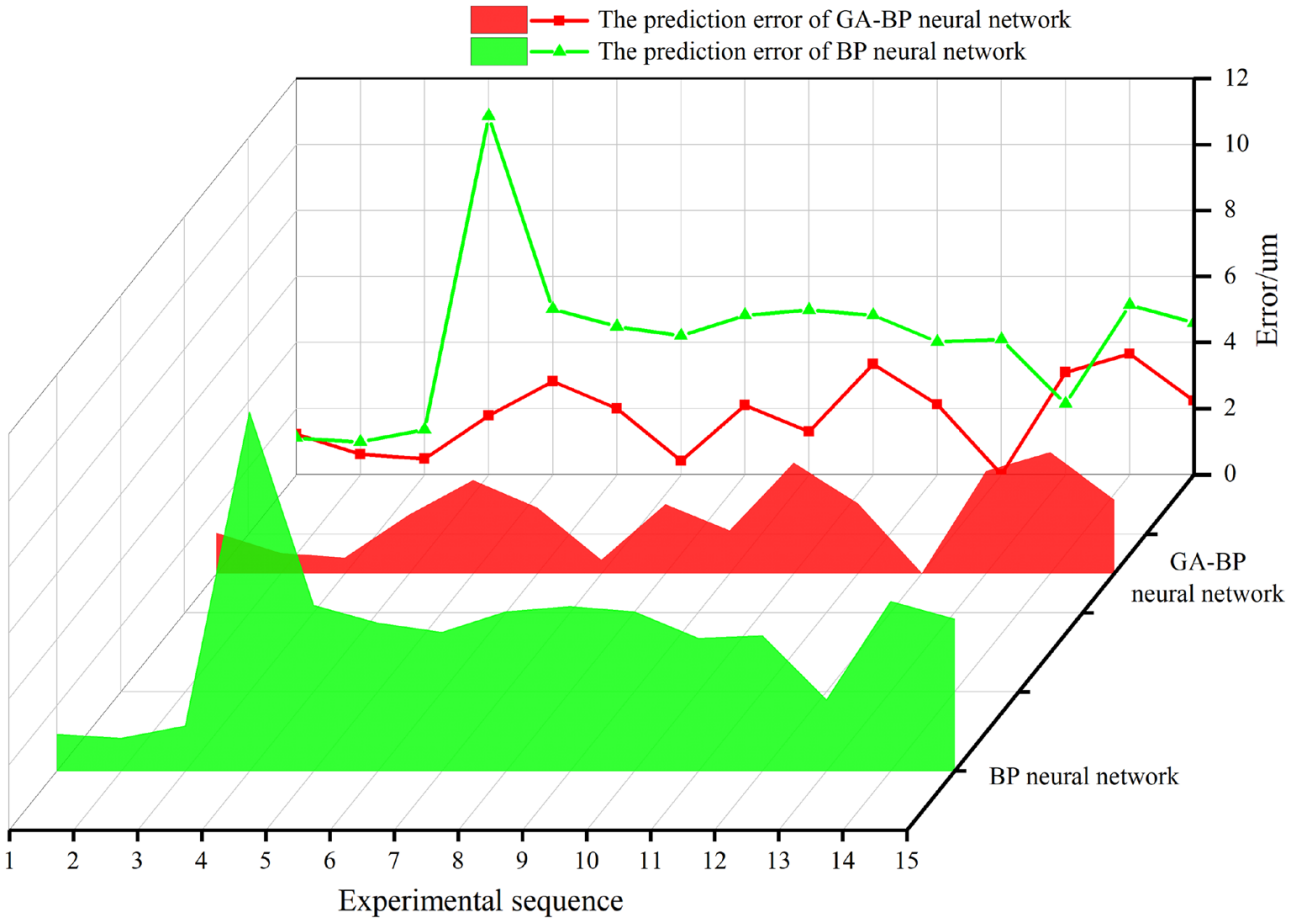
Model prediction error.

The error between the predicted output value and the experimental value based on BP and GA-BP network is shown in Fig. 14. The error analysis of the prediction model is shown in Table 5. The prediction of R^2^ by BP is 0.897 and the prediction of R^2^ by GA-BP is 0.979. The fitting degree of GA-BP model is higher and the prediction effect is better. The prediction error of BP network in the model test of the last 15 groups of data is large, and the prediction errors of MSE, MAE and MAPE are 22.729, 4.172 and 0.072 respectively, as shown in Table 6. After optimization by genetic algorithm, the errors of MSE, MAE and MAPE are greatly reduced to 4.471, 1.811 and 0.031 respectively, indicating that the SMD atomization model based on GA-BP network is successful.

**Table 6:** Atomization model error parameters.

Test model	R^2^	MSE	MAE	MAPE
BP	0.897	22.729	4.172	0.072
GA-BP	0.979	4.471	1.811	0.031

## Conclusion

1. Based on GA-BP model training, the relationship between normalized SMD and normalized input parameters is obtained. SMD decreased with the increase of *Q*_*g*_, showing a positive correlation, while SMD increased with the increase of d and *Q*_*l*_, showing a negative correlation. The relationship between the normalized parameters of model training and the sensitivity of SMD is as follows: *Q*_*g*_ > *d* > *Q*_*l*_.
2. Through the input parameter gas phase flow rate, the liquid phase flow rate, liquid phase inlet diameter and output parameters are trained and tested. The R^2^ predicted by BP model is 0.897, while that of GA-BP is 0.979, which shows strong learning and adaptive performance and high goodness of fit. The mean square error (MSE), average absolute error (MAE) and average absolute percentage error (MPE) of GA-BP prediction are 4.471, 1.811 and 0.031 respectively. Compared with the prediction of BP network, it decreased by 18.258 and 0.041 respectively. GA-BP can effectively improve the prediction accuracy.
3. The weight and threshold of BP neural network are improved by genetic algorithm, the global intelligent adaptability of the network is improved, the convergence of BP network calculation is faster and more accurate, and the droplet size SMD can be predicted quickly. The SMD prediction model of droplet size constructed by GA-BP network greatly improves the prediction speed and accuracy. It is of great significance to quantitatively guide the setting of gas–liquid phase parameters and the improvement of liquid carrying rate in downhole supersonic atomization drainage.

The supersonic atomizing nozzle is used for drainage and gas recovery. The supersonic atomizing is uniform, which effectively reduces the gas–liquid phase velocity slip loss, improves the liquid carrying efficiency and enhances the recovery rate. At present, the new supersonic atomizing nozzle designed by us has been successfully applied to the Xushen gas field block of Daqing Oilfield, and has achieved good results. It can improve the natural gas recovery rate by 4.5–8.6% and alleviate the problem of liquid accumulation near the final stage of oil exploration.

## Supplementary Information

Supplementary Information.

## Data Availability

The datasets generated and analysed during the current study are available in the "supplementary file" repository. The datasets used and analysed during the current study available from the corresponding author on reasonable request. All data generated or analysed during this study are included in supplementary information files.

## References

[1] Shen Y, Luan G (2017). Optimization of coiled-tubing drainage gas recovery technology in tight gas field. Adv. Mech. Eng..

[2] Chunsheng W, Xiaohu W, Qiuying DU (2014). The mechanism study of vortex tools drainage gas recovery of gas well. Adv. Petrol. Explor. Dev..

[3] Ma CB (2014). The efficiency calculation and structural optimization of Eddy composite drainage and gas recovery. Adv. Mater. Res..

[4] Huang B, Li X, Fu C (2019). The mechanism of drainage gas recovery and structure optimization of an internal vortex tool in a horizontal well. J. Nat. Gas Sci. Eng..

[5] Veeken CAM, Belfroid SPC (2011). New perspective on Gas-Well liquid loading and unloading. SPE Prod. Oper..

[6] Chang P, Bai B (2017). An improved method of gas well deliquification using supersonic nozzle. Int. J. Heat Mass Transf..

[7] Surendra M, Falcone G, Teodoriu C (2009). Investigation of swirl flows applied to the oil and gas industry. SPE Proj. Facil. Contract..

[8] Tropea C (2011). Optical particle characterization in flows. Annu. Rev. Fluid Mech..

[9] Chigier N (2006). Challenges for future research in atomization and spray technology: Arthur Lefebvre memorial lecture. Atom. Sprays.

[10] Han H, Wang P, Li Y (2020). Effect of water supply pressure on atomization characteristics and dust-reduction efficiency of internal mixing air atomizing nozzle. Adv. Powder Technol..

[11] Prostański D (2013). Use of air-and-water spraying systems for improving dust control in mines. J. Sustain. Min..

[12] Jian F (2021). Evaluation of application of supersonic atomization drainage technology for low-pressure and low-yield gas wells. Petrochem. Technol..

[13] Ni J, Liu T, Yao L, Xu J (2021). Study on the adaptability of downhole supersonic atomization drainage process. Nat. Gas Oil.

[14] Benjamin, M.A., Mansour, A., Samant, U.G. *et al.* Film thickness, DroPlet size measurements and correlations for large pressure-swirl atomizers. In *ASME/IGTI Conference, Stockholm, Sweden*, 2–5 (1998).

[15] Moon S, Abo-Serie E, Bae C (2009). Air flow and pressure inside a pressure-swirl spray and their effects on spray development. Exp. Therm. Fluid Sci..

[16] Lee H, Lee J, Kim D (2019). Optimization of pre-swirl nozzle shape and radial location to increase discharge coefficient and temperature drop. J. Mech. Sci. Technol..

[17] Amsden, A. A., O Rourke, P. J. & Boter, T. D. A computer program for chemically reactive flows with sprays. Los Alamos National Laboratory report LA-11560-MS (1989).

[18] Lilan H, Qian J, Pan N (2021). Study on atomization particle size characteristics of two-phase flow nozzle. J. Intell. Fuzzy Syst..

[19] Yu H, Chao Z, Liu J (2008). Experimental study of the atomizing performance of a new type of nozzle for coal water slurry. Energy Fuels.

[20] Suh H, Chang S (2008). Effect of cavitation in nozzle orifice on the diesel fuel atomization characteristics. Int. J. Heat Fluid Flow.

[21] Xia Y, Khezzar L, Alshehhi M (2017). Droplet size and velocity characteristics of water-air impinging jet atomizer. Int. J. Multiphase Flow.

[22] Wang, D. *et al.* Study on prediction method of atomized particle size of centrifugal nozzle under variable working conditions. In *Proceedings of the 6th Aerospace Power Joint Conference & the 42nd Technical Exchange Meeting of China Aerospace Third Professional Information Network & 2021 Aero-engine Technology Development High-level Forum (Volume 3)*. (2022).

[23] Yuan HY (2016). Design Theory and Experimental Study of Air Swirling Ejector Nozzle.

[24] Nonnenmacher S, Piesche M (2000). Design of hollow cone pressure swirl nozzles to atomize Newtonian fluids. Chem. Eng. Sci..

[25] Mohanty YK, Mohanty BP, Roy GK (2009). Effect of secondary fluidizing medium on hydrodynamics of gas–solid fluidized bed—Statistical and ANN approaches. Chem. Eng. J..

[26] Zhikai W, Honghua Z, Yibo L (2021). Atomization model of pre-film based on neural network [J/OL]. Adv. Technol..

[27] Liu H, Gong X, Li W (2006). Prediction of droplet size distribution in sprays of prefilming air-blast atomizers. Chem. Eng. Sci..

[28] Kaiser R, Kim S, Lee D (2020). Deep data analysis for aspiration pressure estimation in a high-pressure gas atomization process using an artificial neural network. Chem. Eng. Process.-Process Intensif..

[29] Zhang Y, Xu S, Zhong S (2020). Large eddy simulation of spray combustion using flamelet generated manifolds combined with artificial neural networks. Energy AI.

