# Supplementary material for: Atomized droplet size prediction for supersonic atomized water drainage and natural gas extraction
Source: Sci Rep. 2022 Dec 23;12:22192. doi: 10.1038/s41598-022-26597-x (PMC9789073; doi:10.1038/s41598-022-26597-x)
Supplement: Supplementary file 1 — Supplementary Information. [file 41598_2022_26597_MOESM1_ESM.docx]

Experimental raw data

| *Q_g_* /m^3^ | *Q_l_* / m^3^ | *d*/mm | *D_32_* /μm |
| --- | --- | --- | --- |
| 3000 | 0.6 | 6 | 148.00 |
| 3000 | 1 | 6.6 | 164.92 |
| 3000 | 1.4 | 7.2 | 177.90 |
| 3000 | 1.8 | 7.8 | 188.67 |
| 3000 | 2.2 | 8.4 | 198.28 |
| 3000 | 0.6 | 6.6 | 153.45 |
| 3000 | 1 | 7.2 | 170.87 |
| 3000 | 1.4 | 7.8 | 184.37 |
| 3000 | 1.8 | 8.4 | 195.65 |
| 3000 | 2.2 | 6 | 173.43 |
| 3000 | 1 | 7.8 | 177.34 |
| 3000 | 1.4 | 8.4 | 191.34 |
| 3500 | 0.6 | 6.6 | 108.50 |
| 3500 | 1 | 7.2 | 125.93 |
| 3500 | 1.4 | 7.8 | 139.42 |
| 3500 | 1.8 | 8.4 | 150.70 |
| 3500 | 2.2 | 6 | 128.49 |
| 3500 | 0.6 | 7.2 | 114.46 |
| 3500 | 1 | 7.8 | 132.40 |
| 3500 | 1.4 | 8.4 | 146.40 |
| 3500 | 1.8 | 6 | 125.85 |
| 3500 | 2.2 | 6.6 | 133.93 |
| 3500 | 1 | 8.4 | 139.38 |
| 3500 | 1.4 | 0.6 | 95.54 |
| 4000 | 0.6 | 7.2 | 82.90 |
| 4000 | 1 | 7.8 | 100.84 |
| 4000 | 1.4 | 8.4 | 114.84 |
| 4000 | 1.8 | 6 | 94.30 |
| 4000 | 2.2 | 6.6 | 102.37 |
| 4000 | 0.6 | 7.8 | 89.37 |
| 4000 | 1 | 8.4 | 107.82 |
| 4000 | 1.4 | 6 | 89.99 |
| 4000 | 1.8 | 6.6 | 99.74 |
| 4000 | 2.2 | 7.2 | 108.33 |
| 4000 | 1 | 6 | 82.97 |
| 4000 | 1.4 | 6.6 | 95.44 |
| 4500 | 0.6 | 7.8 | 67.21 |
| 4500 | 1 | 8.4 | 85.66 |
| 4500 | 1.4 | 6 | 67.84 |
| 4500 | 1.8 | 6.6 | 77.58 |
| 4500 | 2.2 | 7.2 | 86.18 |
| 4500 | 0.6 | 8.4 | 74.19 |
| 4500 | 1 | 6 | 60.81 |
| 4500 | 1.4 | 6.6 | 73.28 |
| 4500 | 1.8 | 7.2 | 83.54 |
| 4500 | 2.2 | 7.8 | 92.64 |
| 4500 | 1 | 6.6 | 66.26 |
| 4500 | 1.4 | 7.2 | 79.24 |
| 5000 | 0.6 | 8.4 | 58.64 |
| 5000 | 1 | 6 | 45.26 |
| 5000 | 1.4 | 6.6 | 57.73 |
| 5000 | 1.8 | 7.2 | 67.99 |
| 5000 | 2.2 | 7.8 | 77.09 |
| 5000 | 0.6 | 6 | 33.79 |
| 5000 | 1 | 6.6 | 50.70 |
| 5000 | 1.4 | 7.2 | 63.68 |
| 5000 | 1.8 | 7.8 | 74.45 |
| 5000 | 2.2 | 8.4 | 84.07 |
| 5000 | 1 | 7.2 | 56.66 |
| 5000 | 1.4 | 7.8 | 70.15 |
